# The 23rd Annual Meeting of the Rocky Mountain Virology Association

**DOI:** 10.3390/v16040586

**Published:** 2024-04-10

**Authors:** Ali L. Brehm, Tillie J. Dunham, Samantha M. Pinto, Kaitlynn A. Williams, Kathryn L. Coffin, Molly E. Ring, Oshani C. Ratnayake, Joel Rovnak, Rushika Perera

**Affiliations:** Department of Microbiology, Immunology, and Pathology, Colorado State University, Fort Collins, CO 80523, USA; ali.brehm@colostate.edu (A.L.B.); tdunham@rams.colostate.edu (T.J.D.); kaitlynn.williams@colostate.edu (K.A.W.); kathryn.coffin@colostate.edu (K.L.C.); molly.ring@colostate.edu (M.E.R.); oshani.ratnayake@colostate.edu (O.C.R.); joel.rovnak@colostate.edu (J.R.)

**Keywords:** prions, host–virus interactions, One Health, coronaviruses, arboviruses, vaccines

## Abstract

Located 50 miles west of Fort Collins, Colorado, Colorado State University’s Mountain Campus in Pingree Park hosted the 23rd annual Rocky Mountain Virology Association meeting in 2023 with 116 participants. The 3-day event at the end of September consisted of 28 talks and 43 posters that covered the topics of viral evolution and surveillance, developments in prion research, arboviruses and vector biology, host–virus interactions, and viral immunity and vaccines. This year’s Randall Jay Cohrs keynote presentation covered the topic of One Health and emerging coronaviruses. This timely discussion covered the importance of global disease surveillance, international collaboration, and trans-disciplinary research teams to prevent and control future pandemics. Peak fall colors flanked the campus and glowed along the multiple mountain peaks, allowing for pristine views while discussing science and networking, or engaging in mountain activities like fly fishing and hiking. On behalf of the Rocky Mountain Virology Association, this report summarizes select presentations from the 23rd annual meeting.

## 1. Introduction

**Figure 1:**
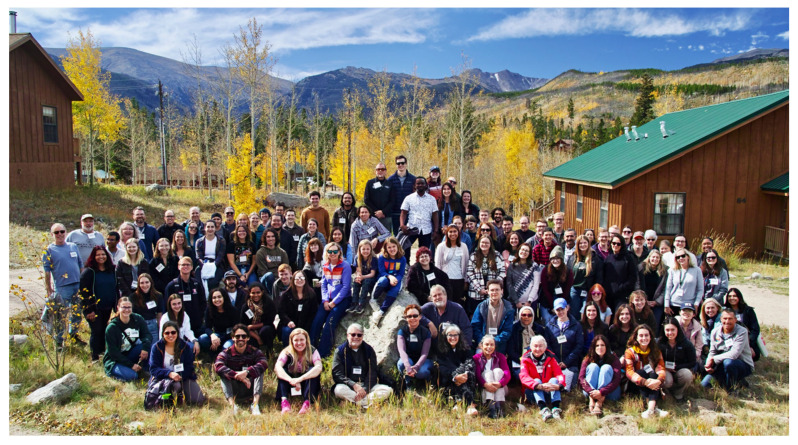
Attendees of the 23rd Rocky Mountain Virology Association meeting.

**Figure 2:**
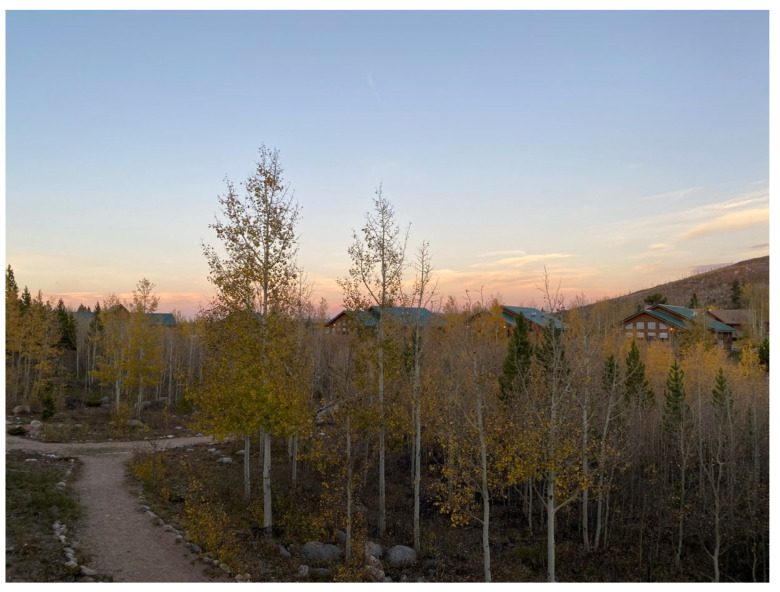
CSU Mountain Campus, showcasing the vibrant aspens that call this area home. Photo by Kathryn Coffin, undergraduate at Colorado State University.

Colorado State University’s Mountain Campus is the host for a wide variety of organizations throughout the year, with their hosting season often capped off with the annual Rocky Mountain Virology Association meeting in late September. Established in 2000, the Rocky Mountain Virology Club (RMVC) has aimed to create a place where virologists at any stage of their career, from undergraduates to senior scientists, can collaborate and share their work among their peers. In 2009, RMVC expanded to include prion biology, and in 2010, the RMVC was renamed Rocky Mountain Virology Association (RMVA) and with the new name came a steady growth in participants that has continued throughout the entire 23-year history of the organization. What once was a meeting of a dozen minds from the Rocky Mountain area has now expanded to over 100 participants from around the country and around the world (Figure 1). This year, 116 participants convened at the colorful campus (Figure 2) from 30 September to 1 October for the conference, which featured five sessions, six invited speakers, 28 oral presentations, and 43 poster presentations.

To kick off the meeting, Dr. Linda Saif from The Ohio State University presented the Randall Jay Cohrs keynote address on a One Health approach with regard to emerging coronaviruses and the importance of global surveillance and international communication. Along with Dr. Saif, both national and international invited speakers included Dr. Catherine Haigh for the Richard A. Bessen lecture on human prion disease in organoids, Dr. Tem Morrison who spoke about arbovirus viremia, Dr. Angela Rasmussen’s presentation on susceptible hosts for emerging viruses, Dr. Anna Cliffe’s talk on the HSV epigenome, and Dr. Sara Sawyer’s presentation on HIV in a new model.

To encourage more lively evening poster presentations, all poster presenters gave lightning talks. These talks, an informal short presentation of less than 5 min and only one PowerPoint slide, could include any information chosen by the presenter. This year, much like previous years, brought laughs and smiles with a gameshow, costumes, and jokes. This year, as every year, the RMVA showcased virology, prion biology, mentorship, and fun. Selected abstracts are presented below.

## 2. Summary of Scientific Sessions

### 2.1. The Randall Jay Cohrs Lecture—Keynote Address

This year’s keynote address was delivered by Dr. Linda J. Saif, from the Center for Food Animal Health Research, Animal Sciences Department, OARDC, College of Food, Agricultural and Environmental Sciences; Veterinary Preventive Medicine Department, College of Veterinary Medicine, The Ohio State University. Dr. Saif presented her research group’s work on “Global disease threats and One Health perspectives on SARS-CoV-2 and emerging coronaviruses of humans and animals”. A devastating and ongoing pandemic was ignited by the precipitous global spread of severe acute respiratory syndrome coronavirus-2 (SARS-CoV-2). It was preceded by the deadly zoonoses of SARS-CoV and MERS-CoV that belong to two species of the genus Betacoronavirus. Most mammalian CoVs, including the endemic human CoVs, likely originated in bat reservoirs, with some infecting intermediate animal hosts (SARS-CoV: civet cats, MERS-CoV: camels) prior to spillover into humans or other animals. Notably betacoronaviruses (betaCoVs) from wild ungulates (cervids) experimentally infect cattle; historically cattle transmitted them to other species (humans, pigs, dogs, poultry). Multiple factors influence interspecies and zoonotic CoV transmission: the environment (e.g., wet markets with animal/human contact); the pathogen (unique features of RNA viruses, dose, stability, transmissibility, etc.); and reservoir-host interactions (receptors, type/frequency of exposure, superspreaders, etc.). Live animal markets were implicated in SARS-CoV and potentially SARS-CoV-2 outbreaks. Besides the high transmissibility of SARS-CoV-2 in humans and the emergence of variants, a major concern for viral persistence is its spillover (reverse zoonoses) and adaptation to animals. SARS-CoV-2 spilled over from humans to pets (cats, dogs, hamsters), farmed mink and various wildlife (white-tailed deer, etc.) with evidence for spillback and secondary transmission to humans. To date 34 host species, many of them wildlife, are susceptible to SARS-CoV-2 natural or experimental infections. A major concern is that if SARS-CoV-2 becomes established in an animal reservoir(s), the virus can persist, mutate and continue to evolve in the new host species, extending its host range and its potential to reinfect humans or other species. Based on historical precedent and the continued circulation of CoVs closely related to SARS-CoV-2 in bats and new host reservoirs, novel coronaviruses will continue to emerge in animals and humans. A One Health approach encompassing global disease surveillance, international collaboration and trans-disciplinary research teams is critical to prevent and control future pandemic threats, such as the WHO projected “Disease X”. All animal studies were performed following guidelines and protocols approved by the Institutional Animal Care and Use Committee of The Ohio State University

### 2.2. Exploring Viral Evolution and Surveillance Methods

Mollie Burton, together with Kirsten Reed, Colin Korte, Tim Wolbers, Samantha Hilty, and Christie Mayo from the Department of Microbiology, Immunology, and Pathology of Colorado State University, presented her work on the prevalence and seasonality of Bluetongue virus on the Front Range of Colorado in domestic ruminants during 2021 and 2022. Mollie emphasized that Bluetongue virus (BTV) is an economically significant pathogen affecting ruminants around the world. Although it is endemic in the US, climate variability has resulted in increased outbreaks for previously naïve populations, likely due to the range expansion of the insect vector, the Culicoides midge. Additionally, only serotypes 3, 6, 10, 11, 12, 13, and 17 are considered established in the US, with serotypes 3, 10, 11, 13, and 17 previously detected in Colorado. The presented work aims to provide current information regarding the seroprevalence and seasonality associated with active infections, and to identify which serotypes are present on the Front Range of Colorado. Serum and whole-blood samples were collected from sheep and cattle along the Front Range of Colorado starting in mid-summer and ending in December. Pan-BTV and serotype-specific RT-qPCR, along with cELISA, were used to evaluate these samples. Site-level seroprevalence and viral RNA prevalence in sheep flocks ranged from 7 to 93% (mean = 47%) and 0 to 67% (mean = 20%), respectively, while cattle herds ranged from 20 to 93% (mean = 56%) and 7 to 40% (mean = 22%), respectively. Of the samples for which serotypes were identified from 2021, BTV-6 and BTV-11 comprised the majority of positive samples, with BTV-6 accounting for 53% of positive sheep samples and 31% of positive cattle samples. For flocks/herds in which serial sampling was performed, most positive cases occurred during the months of September/October, with RNAemia continuing through December. In 2022, site-level seroprevalence in sheep flocks ranged from 16 to 89% (mean = 51%) and from 6 to 100% (mean = 51%). The data from this work highlighted the presence of a previously unreported BTV serotype in Colorado, BTV-6, in addition to expanding upon previous studies examining the prevalence of BTV within sheep and cattle populations in the region. These findings support the continued circulation of BTV in domestic livestock populations, exemplify BTV-6 as a newly established serotype in the state, and will provide data for predictive modeling efforts. All animal studies were performed following guidelines and protocols approved by the Institutional Animal Care and Use Committee of Colorado State University. Funding for this project is provided by USDA-NIFA AFRI grant 2019-67015-28982 as part of the joint USDA-NSF-NIH-BBSRC-BSF Ecology and Evolution of Infectious Diseases Program.

Jebrail Dempsey from the Center for Vector-Borne Infectious Diseases presented her work on Sequencing West Nile Virus Variants Found in Larimer County During Summer 2022. Her research team included Kendra Quicke, Landon Williams, August Luc and Greg Ebel from the same center. Jebrail mentioned that, since its emergence in the United States in 1999, West Nile virus has persistently infected arthropod and avian populations. As its transmission increases, new strains are introduced from acquired mutations. To define the genomic epidemiology of West Nile virus (WNV) in Ft. Collins, CO, and determine patterns of spread between and within transmission seasons, the research team collected and sequenced West Nile Virus from infected mosquito tissues. Mosquito pools were collected as part of ongoing WNV surveillance activities. Up to 50 Culex mosquitoes were homogenized and RNA extracted and screened for the presence of WNV RNA. Jebrail plans to use amplicon-based deep-sequencing to determine consensus sequences of WNV strains. Metadata for each sequence will include trap location, mosquito species and collection date. Phylogeographic studies will be conducted using this data along with data from the past ten years to discern patterns of virus spread within the studied community and population genetics analyses will be conducted to define evolutionary pressures shaping WNV populations. All animal studies were performed following guidelines and protocols approved by the Institutional Animal Care and Use Committee of Colorado State University. The project was funded through T32GM144856 grant.

Tillie Dunham ^1^, together with Tyler Sherman ^1^, Justin Lee ^1^, William Wilson ^2^, Lee Cohnstaedt ^2^, Tavis Anderson ^3^, Kirsten Reed ^1^, Mark Stenglein ^1^, and Christie Mayo ^1^ (Department of Microbiology, Immunology and Pathology, Colorado State University ^1^; National Bio and Agro-Defense Facility, USDA Agricultural Research Service ^2^; National Animal Disease Center, USDA Agricultural Research Service ^3^), presented her work on Bluetongue virus’s search for more space and time in the sequencing archive. Bluetongue virus (BTV) is an economically important arbovirus of domestic and wild ruminants, primarily sheep. BTV is transmitted by various species of Culicoides biting midges and is non-infectious to humans. BTV is an orbivirus with a genome that spans approximately 19,200 bp over 10 linear segments of double-stranded RNA. In addition to random mutation, BTV undergoes rapid evolution through the reassortment of individual segments between coinfecting parental serotypes. To date, there are 29 distinct serotypes of BTV ranging in pathogenicity and infectivity. Whole-genome sequencing (WGS) permits the investigation of genetic diversification and variability rapidly and comprehensively. Tillie mentioned that her study would apply the WGS approach to analyze a large number of BTV genomes. To implement this effectively, she first had to optimize the extraction and library preparation process of BTV. Optimization was necessary as not many protocols are meant for double-stranded RNA. Then, using the optimized protocol, Tillie performed the extraction, library preparation, and sequencing of ~460 BTV cell culture isolates from the Caribbean, Central America, and South Africa. After sequencing, collected data were combined with ~140 whole-blood and vaccine strain samples previously sequenced from California and four other US states, all of which span a collection period of over 50 years. This extensive BTV archive provides a unique dataset to analyze transmission patterns, serotype emergence, and reassortment in a global and temporal context. It will also aid in creating a viral recombination forecasting tool that may help to manage the insect vector populations. No animal or human studies were conducted during the project. This work was funded by the USDA-NIFA AFRI grant number 2019-67015-28982 as part of the joint USDA-NSF-NIH-BBSRC-BSF Ecology and Evolution of Infectious Diseases program.

Natasha Hodges together with Tony Schountz from the Department of Microbiology, Immunology, and Pathology, Colorado State University presented on the Development of Colorimetric LAMP Assay for Field Surveillance Application and Detection of Coronavirus Prevalence. Development of a rapid and extraction-free diagnostic field method for real-time detection of animal infection status is important for resource management in field surveillance. Using such a method can provide real-time prevalence data and reduce the number of euthanized animals for the collection of necropsy samples and laboratory virus isolation. Current standard diagnostic tools such RT-qPCR are not suitable for such work because of the instrumentation and time required for sample preparation. Loop-mediated isothermal amplification (LAMP) assay amplifies nucleic acids using a single tube per reaction and a constant temperature. The use of a portable nucleic acid amplification instrument can be implemented in the field, eliminating the need for PCR-based assays. A heat pretreatment protocol prior to amplification can reduce inhibitors and increased sensitivity. Using these tools and techniques, Natasha plans to develop a field LAMP assay that can be used for real-time coronavirus detection from oral and rectal swab samples collected from rodents. Further, Natasha mentions that this assay will allow us to identify the relatively few infected rodents that will be euthanized for necropsy and collection of samples that will be used for further analysis and virus isolation in the laboratory. No animal or human studies were conducted for this study. The project is funded by a T32 grant.

Kalani M. Williams ^1^ presented her study on the impact of Angolan rousette bat (*Myonycteris angolensis*) foraging site consistency on spillover potential in the Mount Elgon region of Uganda, together with Natalie R. Wickenkamp ^1^, Emma K. Harris ^1^, Benard Matovu ^2^, Betty Nalikka ^2^, Lillian Nalukenge ^2^, Jack-Michael Mutebi ^2^, Aggrey Siya ^2^, Tanya A. Dewey ^3^, Kevin Castle ^4^, Teddy Nakayiki ^5^, Robert M. Kityo ^2^, Julius J. Lutwama ^5^, and Rebekah C. Kading ^1^ (Dept. of Microbiology, Immunology, and Pathology, Colorado State University ^1^; Zoology, Makerere University ^2^; Biology, Colorado State University ^3^; Wildlife Veterinary Consulting LLC ^4^; Entomology, Uganda Virus Research Institute ^5^). According to her presentation, Angolan rousette bats (*Myonycteris angolensis*) are a potential reservoir for viruses that could be of importance to human/livestock health. Her preliminary data suggested these bats may carry paramyxoviruses and rhabdoviruses. To better understand the potential for viral spillover, bat habitat usage must be elucidated. In this study, Kalani and her team used GPS tracking to ascertain the foraging sites of *M. angolensis* within the Mount Elgon region of Uganda. Utilizing GIS spatial applications, Williams et al. determined if these foraging sites were significantly associated with particular landscape features. GPS data were acquired by suturing GPS units onto bats and taking fixes during periods of high activity over the course of five days in both dry and wet seasons of 2023. Using kernel density algorithms to determine foraging hotspots from the distribution of GPS points, preliminary data suggest foraging hotspots are significantly closer to rivers/streams and protected areas and significantly further from human settlements than would be expected if their distribution was random. Using multi-night data, it was evident that specific foraging locations were visited by multiple bats from the colony and by the same bats over consecutive nights. The primary exception to this is one male who flew up to 170 km every other night during the study period. Foraging ranges were fairly consistent in forested/protected areas across seasons, but different foraging locations were identified in populated urban/agricultural sites in wet versus dry seasons. These results have implications for the impact of seasonality on potential for viral spillover via changes in bat foraging behavior. This work was funded by the US Department of Defense, Defense Threat Reduction Agency HDTRA1-19-1-0030. Additionally, this work is supported by the National Science Foundation Graduate Research Fellowship Program under Grant No. 006784. Any opinions, findings, and conclusions or recommendations expressed in this material are those of the author(s) and do not necessarily reflect the views of the National Science Foundation. All animal studies were performed following guidelines and protocols approved by the Institutional Animal Care and Use Committee of Colorado State University.

### 2.3. Developments in Prion Research

Alyssa J. Block ^1^, along with Diana C. Lowe ^1^, Xutong Shi ^1^, Joseph P. DeFranco ^1^, Julianna L. Sun ^1^, Jenna Crowell ^1^, Sehun Kim ^1^, Jifeng Bian ^1^, Maria Nöremark ^2^, Dolores Gavier-Widen ^2^, Sylvie L. Benestad ^3^, and Glenn C. Telling ^1^ (^1^ Department of Microbiology, Immunology, and Pathology, Colorado State University, United States; ^2^ National Veterinary Institute, Sweden; ^3^ Norwegian Veterinary Institute, Norway), explained her work on the adaptation of Nordic chronic wasting disease isolates. Chronic wasting disease (CWD) is a highly contagious prion disease affecting cervids in North America (NA) and, more recently, Europe. The characterization of emergent CWD in Norway, Sweden, and Finland indicates that these isolates differ from NA CWD. Nordic CWD isolates are more diverse than stabilized NA CWD strains and are non-lymphotropic, with the exception of CWD in Norwegian reindeer. Studies of Nordic CWD in gene-targeted (Gt) mice, which express physiological levels of cervid PrP with either glutamine (Q) or glutamate (E) at residue 226, recapitulate the lymphotropism of natural CWD isolates. Transmission studies of the second Norwegian moose CWD isolate (M-NO2) found that M-NO2 preferentially infects GtQ mice and is non-lymphotropic. However, transmission through GtE mice resulted in the isolation of a strain that more efficiently transmits to the E background and became lymphotropic, resembling NA CWD isolates. To investigate if other non-lymphotropic Nordic CWD isolates undergo a similar adaptation process during serial passage in Gt mice, lymphoid tissue was evaluated for presence of PrP^Sc^. A Western blot of spleen homogenates from three passaged isolates—a Norwegian red deer, Norwegian moose, and Swedish moose—revealed the presence of PrP^Sc^, indicating the acquisition of lymphotropism during serial transmission. Interestingly, these isolates also share a distinct PrP deposition pattern in the dentate gyrus, which is overwhelmingly observed when passaged in the GtE background. Combined, these data suggest the adaptation of diverse, unstable Nordic CWD isolates towards a lymphotropic CWD strain facilitated by passage in the E background, potentially leading to an adaptation event similar to M-NO2. This work was funded by NIH NINDS R35 NS132226-01, R01 NS109376-05, and R01 NS121682-03. All animal studies were performed in accordance with the Guide for the Care and Use of Laboratory Animals, and procedures were approved by the Colorado State University Institutional Animal Care and Use Committee.

Kaitlyn Forrest, along with Erin McNulty, Joseph Westrich, and Candace Mathiason (Department of Microbiology, Immunology, and Pathology, Colorado State University), investigated the correlation between circadian rhythm and prion infection. Circadian rhythms are endogenous oscillations of physiological functions entrained to cyclic environmental cues. The molecular clock is composed of a transcriptional/translational feedback loop (TTFL) of a family of transcription factors known as core clock genes which cyclically regulate their own expression and the expression of other genes (clock-controlled genes). Prnp encodes the mammalian prion (PrPC) protein, which undergoes misfolding into a pathogenic and transmissible isoform (PrPSc) in prion disorders. To date, the native function for PrP has yet to be definitively identified. Previous work has alluded to the genetic deletion of Prnp altering behavior-level circadian phenotypes, yet the relationship between the molecular clock and PrPC is unknown. To further explore this, Kaitlyn collected tissue from mice (wild-type C57bl/6j, FVB-KO (Prnp-KO), and Tg (CerPrP-E226)5037+/− (Prnp over-expressing, herein 5037) harvested at indicated times. She described, for the first time, the oscillations in the expression of core clock genes and Prnp in the brains of transgenic mice expressing variable concentrations of PrPC. She found that, compared to C57bl/6j, 5037 displayed drastic alterations in oscillation patterns of clock gene expression, while FVB-KO displayed more mild differences in clock gene rhythms. Notably, Prnp expression was arhythmic in C57bl/6j but rhythmic in 5037 mice. In addition, Forrest et al. described oscillations in immune cell populations in the spleen and lymph nodes of FVB-KO and 5037 transgenic mice. Their results indicate that levels of Prnp expression do impact molecular and cellular circadian rhythms, indicating a role of native Prnp within the mammalian clock and, conversely, implying a role of circadian rhythms in prion disorders. This work was funded by NIH NIAID R01AI156037 and NIH NIAID R01AI112956. All animal studies were performed following guidelines and protocols approved by the Institutional Animal Care and Use Committee of Colorado State University.

Cathryn L. Haigh (Laboratory of Neurological Infections and Immunity, National Institute of Allergy and Infectious Diseases) presented research on developing organoids for studying prion infection. Prion diseases are infectious neurodegenerative diseases affecting humans and animals. They are caused by prions: misfolded conformers of a cellular protein called the prion protein (PrP) that can recruit and convert more PrP molecules into prions in a self-propagating cycle. Eventually, this cycle overwhelms the brain, causing neuronal dysfunction and death. However, the mechanisms that lead to eventual death are largely unknown. To better understand human prion disease pathogenesis, the authors developed cerebral organoid models of acquired and genetic prion diseases. To model infectious disease, organoids can be infected with different types of prions and faithfully propagate the original prion. For genetic disease, mutations that pre-dispose individuals have been obtained from carrier donors or by CRISPR-Cas9 engineering. Studies using these models have shown electrophysiological dysfunction both as a result of infection and due to mutation within the PrP gene. Further investigation has identified the important pathological events underpinning these dysfunctions, including cytosolic oxidative stress, shifted cellular energy pathways, and damaged cytoskeletal architecture. The data observed support the notion that changes occur both due to the loss of PrP function and also because of a gain or corruption of function of abnormal PrP. Together, these approaches provide a window into the changes occurring in human brain tissue that cause degeneration during prion disease and the role of PrP function in facilitating such changes. This work was funded by NIAID IRP. All animal and human tissue studies were performed following guidelines and protocols approved by the Institutional Animal Care and Use Committee of RML (NIAID) and the National Institutes of Health (NIH), Office of Human Subjects Research Protections (OHSRP) Institutional Review Board (IRB), respectively.

M. L. Tyer, along with Julie Sun, Joe DeFranco, and Glenn Telling (Department of Microbiology, Immunology, and Pathology, Colorado State University), presented their research on polymorphisms in chronic wasting disease. Polymorphic variations are known to impact the pathogenesis of prion diseases in various hosts and their susceptibility to different prion strains, including chronic wasting disease (CWD) in cervids. Using gene-targeted (Gt) mice expressing elk or deer PrP, Tyer et al. previously demonstrated that amino acid variation of glutamine or glutamic acid at codon 226 affects various aspects of disease pathogenesis, including the selection of distinct strain conformers. Expanding on this concept, they sought to characterize additional polymorphisms described in CWD-susceptible cervids. This includes a substitution of lysine for glutamine at codon 109 associated with abnormal CWD presentation in Scandinavian moose, polymorphisms in the N-terminal signal peptide region that are correlated with altered susceptibility, and polymorphisms in other key regions of the mature prion protein, such as a deletion that results in four octapeptide repeats instead of five. To investigate the effect of these alterations, Tyer et al. utilized a cervid prion cell assay and Western blotting for in vitro characterization. This cell-based screening will help us select polymorphisms of the greatest consequence that can be used to develop new gene-targeted mice for in vivo study. This work was funded by T32GM144856, 5R01NS121682, 5R01NS109376, PO1-0011877A, and 5P01AI077774. All animal studies were performed following guidelines and protocols approved by the Institutional Animal Care and Use Committee of Colorado State University.

Xutong Shi ^1,2^, along with Julianna L. Sun ^1^, Jifeng Bian ^1^, Sehun Kim ^1^, Jenna Crowell ^1^, Bailey Webster ^1^, Emma Raisley ^1^, Erin Flaherty ^1^, and Glenn C. Telling ^1,2,^* (^1^ Prion Research Center (PRC), Department of Microbiology, Immunology and Pathology; ^2^ Program in Cell and Molecular Biology, Colorado State University), provided updates on the features of prions causing chronic wasting disease. Since 2016, emergent forms of CWD have been detected in free-ranging reindeer (Rangifer tarandus), moose (Alces alces), and red deer (Cervus elaphus) from Norway, Finland, and Sweden. These previous studies showed that gene-targeted (Gt) mice which regulate the physiological expression of elk or deer prion protein (PrP) using control elements of the mouse PrP gene (Prnp) are susceptible to Nordic and North American CWD prions, and that the propagation of distinct CWD strains is controlled by naturally occurring amino acid differences at residue 226 of PrP. Using this platform, Shi et al. showed that Nordic CWD prions exhibit considerable strain diversity and that their properties are different from strains causing CWD in North America. Here, they comprehensively assessed the features of prions causing CWD in Norwegian red deer. The properties of these emergent prions, as assessed by susceptibility in Gt mice of different cervid PrP genotypes, Western blotting, neuropathology, and in vitro amplification platforms, are distinct from those causing disease in other Nordic or North American cervids. While the propagation of Norwegian red deer CWD prions is facilitated by the expression of glutamate at residue 226, transmission and in vitro amplification are relatively inefficient compared to other CWD strains, resulting in unusually high frequencies of subclinical disease after long incubation times, despite the presence of abundant CNS prion accumulation in the brains of Gt mice. Our findings are consistent with a growing portfolio of emergent CWD strains in Nordic countries which contrasts the relatively consistent properties of CWD in North American cervids. This work was funded by NIH grant 1RO1NS109376-01. All animal studies were performed following guidelines and protocols approved by the Institutional Animal Care and Use Committee of Colorado State University.

### 2.4. Investigating Arboviruses and Their Vectors

Shelby Cagle ^1^ presented their work with Cheyenne Schad ^2^, Aidan Wolfe ^2^, Halley Pucker ^2^, and Nicole Kelp ^1^ (^1^ Colorado State University, Department of Microbiology, Immunology, and Pathology; ^2^ Colorado School of Public Health) on measuring risk perceptions and the efficiency of place-based communication for West Nile virus in Colorado. Interdisciplinary approaches which consider ecological, social, and human and animal health facets are critical for tackling vector-transmitted diseases. West Nile virus (WNV) is one such disease that transmits to birds, horses, people, and other mammals via bites from infected mosquito vectors. Across Colorado, public health practitioners and researchers are working alongside community members to characterize prevalent risk perceptions and to determine effective messaging strategies that agencies can employ to convey WNV risk. To aid in these efforts, a survey based on the Health Belief Model was developed and deployed to measure individuals’ risk perceptions and personal protective behaviors regarding WNV. Cagle et al. tested four different messages about WNV to assess how these impacted individuals’ risk perceptions and behaviors. Our results show that heat maps of local regions’ WNV-positive mosquito pools and that infographics about WNV placed at trailheads had the most statistically significant increases in individuals’ perceived severity and planned protective behaviors regarding WNV. All interventions tested correlated with increased risk perceptions to WNV. To further test the applications of this finding, they are implementing a citizen science approach to assess how individuals’ risk perceptions correlate with actual risk, as measured by proximity to WNV-positive mosquitoes across Colorado. Results from these ongoing studies were presented. No animal or human studies were performed.

Kathryn Coffin, with Michelle Savran, Claire Stewart, Brian Foy, and Ashley McGrew of the Department of Microbiology, Immunology, and Pathology, presented her work on the use of ivermectin-coated bird feed to control the spread of West Nile virus. West Nile virus (WNV) is a flavivirus vectored by Culex mosquitoes that can cause mild-to-severe disease in humans, with about 0.67% of cases resulting in neuroinvasive disease. People who are immunocompromised, such as the elderly or very young children, are particularly at risk for developing severe symptoms from WNV. One of the main reservoir hosts for this virus is birds, which makes them an ideal target for controlling the spread of WNV. The development and optimization of ivermectin (IVM)-coated bird seed target the vector population and prevent mosquitoes from spreading the virus to humans. When mosquitoes pick up an ivermectin-dosed blood meal, the IVM binds to the glutamate-gated chloride ion channels in the nervous system, leading to paralysis and death. Wild-caught house sparrows, pigeons, and a laboratory colony of zebra finches acted as model birds for these experiments optimizing the safest and most effective concentration of ivermectin in the seed. The parasite load in the wild birds was also evaluated to determine if any parasites affected by ivermectin were present, so as to observe if there was any positive health effect for the birds. It is important to know for future studies how IVM could affect the parasite load of wild birds feeding on seed. Serum from birds fed seed with a 100 mg/kg concentration of ivermectin showed significant insecticidal activity in *Culex tarsalis* mosquitoes while also remaining safe for birds to consume as their sole food source over a 7-day period. All animal studies were performed following guidelines and protocols approved by the Institutional Animal Care and Use Committee of Colorado State University. This study was funded with a grant from the NIH-NIAID.

Kaleb Davis ^1^, with Natalie Wickenkamp ^1^, Julius Stuart ^3^, Alec Jones ^4^, Margaret Yates ^2^, Elisa Thrasher ^2^, Arielle Glass ^1^, Ashlyn Chen ^2^, Christopher Snow ^2,3^, and Rebekah C. Kading ^1^ (Dept. of Microbiology, Immunology, and Pathology, Colorado State University ^1^; Dept. of Biochemistry and Molecular Biology, Colorado State University ^2^; Dept. of Chemistry, Colorado State University, Colorado State University ^3^; Dept. of Biomedical Engineering, Colorado State University ^4^), presented his data on using crystals in mosquito movement research. Arboviral diseases have been and continue to be one of the most pertinent public health problems of the modern world. While we know much about many of the diseases and vectors, there remains a wide dearth of knowledge about how these diseases are spread in the environment. The absence of knowledge is in part due to the lack of information regarding vector movement. The current method of gathering these data is predominantly centered on the application of topical fluorescent dyes to the vector’s surface to track movement. This mark–release–recapture framework is limited in the number of unique dyes and difficulty in application, which may affect the distance of travel and survivability. In order to address this gap in knowledge and the limitations of current technology, this group both developed and piloted a novel microcrystal-based technology for the tracking of mosquitoes in the field. The microcrystals used in this application readily take up DNA when in solution, are small enough to be eaten by larval Culex mosquitoes, and protect taken-up DNA from a number of environmental factors including the mosquito midgut environment. By having these crystals take up DNA with a unique sequence, Davis et al. were able to trace captured mosquitoes back to their heritage site. As such, they successfully recovered barcodes from 53 wild-caught mosquito pools and showed that the barcodes persisted for 5 weeks in the environment. In addition, they demonstrated that the crystals had minimal, if any, impact on the survivability of the mosquitoes in the lab and began dose optimization. No animal or human studies were performed. This research was supported by NIH/NCATS Colorado CTSA Grant Number UL1TR002535 and NIH R21 AI146740. Its contents are the authors’ sole responsibility and do not necessarily represent official NIH views. This work was also supported in part by Animal Health and Disease Grant No. 2021-01/Project Accession No. 1027096 from the USDA National Institute of Food and Agriculture.

Ashley Freedman ^1^, with Gabriela Ramirez ^1,2^ and Karen Dobos ^1^ (^1^ Department of Microbiology, Immunology, and Pathology at Colorado State University; ^2^ Cellular and Molecular Biology at Colorado State University), gave a poster presentation about the molecular mechanisms associated with diapause in *Anopheles stephensi* mosquitoes. Mosquitoes act as pollinators, play an important role in the food chain, and serve as vectors of diseases, which juxtapose them as a keystone species and as the deadliest animal in the world. Aedes, Culex, and Anopheles mosquitoes are vectors of pathogenic viruses such as Zika, dengue, chikungunya, and the parasite Plasmodium, which causes malaria. Some of these vectors co-circulate within the same region, increasing the potential of epidemics and significantly challenging the public health infrastructure in developing countries. This study examined *Anopheles stephensi*, a mosquito native to Asia that has recently expanded to Eastern Africa. Freedman et al. sought to understand the molecular mechanism by which they are able to adapt to different environments. Diapause, a biological dormancy state, allows mosquitoes to survive in unfavorable conditions. They hypothesize that as mosquitoes enter diapause, their metabolic rate fluctuates and lipid production is significantly increased. Lipids play important roles in organisms, such as energy storage and transport in cell membranes, as well as hormone signaling. These key factors of lipids may play a role in their survival. Therefore, it is crucial to assess the lipid metabolomic plasticity of this vector at each life stage. In this study, the authors examined the expression levels of two lipid-producing enzymes: fatty acid synthase and ceramide synthase. They performed an RT-qPCR assay to detect the expression levels of these genes during the adult life stage in a diapause-induced colony compared to a normal colony. They sought to understand the roles of these lipid-producing enzymes and how fluctuation in their expression level can alter diapause success. No human or animal studies were performed. Funding was provided by TO C07 Development of a Cryopreservation Process for Mosquito Vectors of Human Pathogens.

Emily Gallichotte, alongside Greg Ebel (Department of Microbiology, Immunology, and Pathology, Colorado State University), presented a poster on the creation of COMET: vector competence experimental testing, a database for meta-analyses of vector competence. Many recently emerging pathogens are arboviruses, with over one-third of the world’s population at risk for infection. Laboratory-based mosquito infection experiments are often the best way to measure vector competence, and arbovirus–mosquito experimental testing data are critical to understand outbreak risk. There are challenges with experimental studies, and despite having been collected and reported for a large range of vector–pathogen combinations, the terminology is inconsistent, records are scattered across studies, and accompanying publications often share data with insufficient detail for meta-analyses or synthesis. There is a need for a vector competence data standard for reporting, which aligns with a broad effort across scientific disciplines to preserve data for future use, recover existing data that may be unsearchable, and establish open principles for harmonizing those data to better leverage the effort of the larger community of research. Gallichotte et al. recently published a minimum data standard for vector competence experiments which strikes a balance between completeness and labor-intensiveness, with the goal of making these important experimental data easier to find and reuse, without much added effort for those generating the data. They are building a vector competence experimental testing (COMET) database compiling standardized data on the infection, dissemination, and transmission of mosquito-borne viruses. They will perform meta-analyses to decompose extrinsic (temperature, other unaccounted-for experimental variability) and intrinsic (mosquito-omics, viral-omics) drivers, with a focus on mosquitoes and flaviviruses. They will describe the database structure, data types and formatting, and preliminary meta-analyses. No human or animal studies were performed. Funding is through the National Science Foundation (BII 2213854).

Olivia Martinez, with Emma Harris, Shelby Cagle, and Rebekah Kading (Department of Microbiology, Immunology and Pathology, Colorado State University), presented results on the effects of temperature change on the oviposition and progeny viability of *Aedes aegypti* and *Culex tarsalis* mosquitoes. Temperature is known to affect the transmission efficiency of mosquito-borne viruses, particularly those spread by *Aedes aegypti* and *Culex tarsalis* mosquitoes. Investigating how environmental changes impact *Aedes aegypti* and *Culex tarsalis* fecundity will inform future action for vector control and subsequent disease mitigation. Their preliminary data showed impaired egg deposition when Rift Valley fever virus-carrying adult *Ae. aegypti* mosquitoes were exposed to temperatures varying from typical environmental conditions. Therefore, Martinez et al. investigated the relationship between altered temperatures, oviposition rates, and progeny viability within uninfected blood-fed *Ae. aegypti* and *Cx. tarsalis* mosquitoes. They hypothesized that temperature variation would negatively impact egg viability, deposition rates, and offspring development. Blood-fed female mosquitoes (n = 50) were housed individually at lower (18 °C), standard (28 °C), or higher (32 °C) rearing temperatures. Egg production was assessed by quantifying deposited eggs in comparison to withheld eggs, obtained by ovarian dissections. Deposited egg hatch rates were recorded to determine offspring viability. Data collection is ongoing, but preliminary data showed increased developmental rates in *Ae. Aegypti* at 32 °C when compared to standard temperature. Understanding the relationship between mosquito fecundity and temperature is of great importance for anticipating infectious disease dynamics in a complex and shifting global environment. Olivia is a MARC Scholar funded by a grant from the National Institute of General Medical Sciences of the National Institutes of Health: T34GM140958. No human or animal studies were performed.

Austin J. Mejia ^1^, alongside Teddy Nakayiki ^2^, Julius J. Lutwama ^2^, Fred Ssenfuka ^2^, George Ongodia ^2^, Kivumbi Brian^2^, and Rebekah C. Kading ^1^ (^1^ Center for Vector-borne Infectious Diseases, Dept. of Microbiology, Immunology, and Pathology, Colorado State University; ^2^ Department of Arbovirology, Emerging, and Re-emerging Infections, Uganda Virus Research Institute, Entebbe, Uganda), presented his research on *Ae. aegypti* and other mosquito species cohabitating in the Chekwoputoi cave, Uganda. *Aedes aegypti* is a major mosquito vector of globally significant human pathogens. *Ae. aegypti* can transmit viruses such as dengue (DENVs), Zika, chikungunya, and yellow fever. *Ae. aegypti* exhibits a complex genetic structuring among populations in Africa. Significant knowledge gaps remain pertaining to the sylvatic larval habitats of *Ae. aegypti*. The authors opportunistically collected mosquito larvae (n = 113) from a rock pool at the entrance to Chekwoputoi cave located in the Kween District, Uganda. This cave is the known roosting site for a large colony of the African sheath-tailed bat, *Colura afra,* and is regularly utilized by domestic and other wild mammal species. Mosquitoes were reared to adults at the Uganda Virus Research Institute and morphologically identified. This collection comprised 7 species: *Ae. aegypti formosus* (n = 5), *Anopheles rhodesiensis*, and 5 additional *Culex* and *Aedes* species. Species identifications were confirmed using molecular techniques and documented using high-resolution photography. These observations represent a unique ecological insight into the larval habitat and mixed-species larval community of medically important mosquito species in Uganda. They hope to utilize this information to understand mosquito vector ecology in this poorly studied area and how these vectors cohabitate with each other. This study was funded by IMSD. No animal or human studies were performed.

Hunter Ogg, along with Natasha Hodges, Liz Mielke, Rebekah Kading, and Corey Campbell (Department of Microbiology, Immunology, and Pathology, Colorado State University), presented his research on the transcriptome reference map of adult *Culex tarsalis* ovaries. Their group is investigating the effects of the Rift Valley fever virus (RVFV) on vectors (mostly notably *Culex tarsalis* and *Aedes aegypti* mosquitoes) to understand the origins and spread of outbreaks. There is evidence that they can pass RVFV to their offspring through transovarial transmission. The animal reservoir of RVFV in nature has not been identified. Until then, mosquitoes could serve as an epizootic reservoir of the virus between outbreaks. Blood-fed adult *C. tarsalis* mosquitoes were held for 7 days until eggs were laid. A total of 20 ovaries were harvested for each sample. Single-cell RNA sequencing was performed using 3′ 10X Genomics technology, and Illumina sequencing was performed to enrich for mRNA transcripts. The resulting data were analyzed using Cellranger3, Seurat, and SingleCellTK libraries to identify cell types based on the transcriptome and isolate genetic markers. Sequencing data were correlated with a list of putative genes identified through BLAST. Ovarian cell transcriptomes mapped onto nine distinct clusters. Prominent proteins with significant expression differences between ovarian cell types, such as oskar, SUMO1, and ribosomal proteins, are well characterized and in line with previous arthropod studies. These were used to classify clusters such as germ and follicle cells. While clustering findings were robust between samples, there was heterogeneity among marker genes. This preliminary information is a baseline for genetic expression in *C. tarsalis* ovaries and allows for subsequent studies of differential expression in infected mosquito ovaries. This information could eventually inform public health responses to a disease whose transmission and reservoirs are poorly understood. This study was funded by the NIH and USDA. No animal or human studies were performed.

Samantha M. Pinto ^1^^,2,3^, along with Oshani C. Ratnayake ^1,2,3^, Paul S. Soma,^1,2,3^ Suad Elmegerhi ^1,2,3^, Venugopal Pujari ^2,3^, Dean Crick ^2,3^, Elizabeth A. McGraw ^4^, and Rushika Perera ^1,2,3^ (^1^ Center for Vector-borne Infectious Diseases; ^2^ Center for Metabolism and Infectious Diseases; ^3^ Dept. of Microbiology, Immunology, and Pathology, Colorado State University; ^4^ Department of Biology, Center for infectious Disease Dynamics, The Huck Institutes of the Life Sciences, Pennsylvania State University, State College, PA), presented her research on triacylglycerol dynamics: determining the role of TGs in the lifecycles of arboviruses in *Aedes aegypti*. Mosquito-borne diseases account for ~17% of the global disease burden, plaguing more than half of the world’s population. Dengue (DENV), Zika (ZIKV), and chikungunya (CHIKV) viruses are arboviruses primarily transmitted by *Aedes aegypti* mosquitoes. This team’s previous studies have shown that infection with these viruses causes significant changes to the lipid metabolome of the mosquito. Using liquid chromatography–mass spectrometry, they discovered a substantial increase in the glycerolipid class, specifically triacylglycerols (TGs), 7 and 14 days post-infectious blood meal with DENV, ZIKV, or CHIKV. Notable differences between TG level trends in flaviviruses (DENV, ZIKV) and alphaviruses (CHIKV) were observed. They hypothesized that TG levels increase in response to virus infection and may play a significant role in viral replication, dissemination, and transmission in the vector. Alternately, TG levels may influence the mosquito immune response to infection. Additionally, TG requirements between flaviviruses and alphaviruses may differ during the infectious cycle. To test this hypothesis, they inhibited TG synthesis in a mosquito host using a chemical inhibitor followed by initial infection with DENV, serotype 2 (DENV2). Preliminary data showed that the inhibition of TG synthesis decreased the viral titer in the mosquito. This was observed in vitro in C6/36 *Aedes albopictus* larval cells as well as in vivo with *Ae. aegypti* mosquitoes. Their current efforts are expanding these inhibitor studies to both ZIKV and CHIKV, as well as determining how the inhibition of TG synthesis impacts viral replication in the midgut, dissemination to the body, and transmission via the saliva. This study was funded by NIH-NIAID grant R01AI151166. No animal or human studies were performed.

Brian Prince, along with Tran Zen B Torres, Kalvin Chan, and Claudia Rückert (Department of Biochemistry and Molecular Biology, University of Nevada, Reno), presented his research investigating the alternative antiviral functions of Dicer-2 in the West Nile virus mosquito vector *Culex quinquefasciatus*. These mosquitoes transmit many pathogens of human importance, including West Nile virus and Saint Louis encephalitis virus. Improving the understanding of mosquito immune pathways has the potential to translate into novel vector control methods. Recognizing the presence of infecting viruses is the first step in eliciting an immune response. Long double-stranded RNA (dsRNA), which can be generated during virus replication, is generally foreign to animal cells and acts as a pathogen-associated molecular pattern. The major antiviral response triggered by dsRNA in mosquitoes is RNA interference (RNAi)—a sequence-specific response which targets complementary viral RNA for degradation and is initiated by the protein Dicer-2. However, here, the authors aimed to determine if dsRNA sensing by Dicer-2 triggers a more global antiviral immune response in a sequence-independent manner. To this end, they generated a CRISPR/Cas9-mediated *Cx. quinquefasciatus* cell line with a knockout of Dicer-2. They validated their cell line with Sanger sequencing and also demonstrated the loss of RNAi function. In addition, in a protein pulldown using poly(I:C), a generic dsRNA mimic, Dicer-2 failed to bind poly(I:C) in their knockout cells compared to wild-type cells. Their approach also enabled identifying Dicer-2 binding partners that may be involved in downstream immune signaling. Using their cell line, they were able to determine how the dsRNA sensing of Dicer-2 contributes to the control of virus replication, independent of RNAi. Overall, they aimed to establish a more complete picture of the mosquito immune system, which may inform future transmission control strategies. This study was funded by Nevada INBRE. No animal or human studies were performed.

Oshani C. Ratnayake ^1^, along with Paul S. Soma ^1^, Nunya Chotiwan ^1,2^, Samantha Pinto ^1^, Irma Sanchez Vargas ^1^, Barbara Graham ^1^, Kaitlyn Dirks ^1^, Elizabeth McGraw ^3^, and Rushika Perera ^1^ (^1^ Center for Vector-borne Infectious Diseases, Dept. of Microbiology, Immunology and Pathology, Colorado State University, Fort Collins, CO; ^2^ Chakri Naruebodindra Medical Institute, Faculty of Medicine Ramathibodi Hospital, Mahidol University, Thailand; ^3^ Department of Biology, Center for infectious Disease Dynamics, The Huck Institutes of the Life Sciences, Pennsylvania State University, State College, PA), presented her research on arboviral infections and how they induce metabolic remodeling in mosquito vectors. Dengue, Zika (flaviviruses), and chikungunya (alphaviruses) viruses are among the most aggressive arboviruses spread by the bite of an infected *Ae. aegypti* mosquito. These viruses cause a spectrum of symptoms, from self-limiting febrile diseases to fatal hemorrhagic fever, neurological disorders, or chronic arthritis. In the absence of successful antivirals or effective vaccines, these viruses remain a significant health burden. Using systems biology, the authors showed that these three arboviruses significantly alter the metabolome of *Ae. aegypti* during infection. They observed key changes in bioactive fatty acids, sphingolipids, and glycerolipids, as well as changes in structural glycerophospholipids and sterols. Both common and distinct metabolic alterations were observed between arboviruses, indicating diverse virus–vector interactions. A key pathway of interest is glycerolipid metabolism. Glycerolipids consist of triacylglycerols (TAGs), monoacylglycerols (MAGs), and diacylglycerols (DAGs). TAGs play a significant role in energy metabolism and can be stored in body fat as an energy reserve or be transported to the ovaries for vitellogenesis. The authors observed an increased abundance of TAGs during infection with all arboviruses. Alternately, MAGs and DAGs were only increased early during infection. This suggests that TAGs in the blood meal or in lipid reserves are hydrolyzed early during viral replication to generate more MAGs and DAGs, which are mobilized to cater to the demands of viral replication. Higher levels of TAGs are also achieved by the conversion of MAGs and DAGs via diacylglycerol o-acyltransferase activity as a response to infection. They investigated the interaction between glycerolipid metabolism and vector immunity and its impact on infection success using genetic manipulation tools and inhibitors to probe this metabolic pathway. Additionally, they investigated how Wolbachia infection modulates this pathway in the presence and absence of viral infection. Their goal was to identify metabolic choke points in the mosquito vector that can be effective in vector control. This study was funded by NIH-NIAID grant R01AI151166. No animal or human studies were performed.

Molly Ring, along with Claire Stewart, Chilinh Nguyen, Shelby Cagle, Jenna Randall, Halley Pucker, Brian Foy, and Nicole Kelp (Dept. of Microbiology, Immunology, and Pathology, Colorado State University, Fort Collins, CO), presented her research on the efficacy of ivermectin-treated bird feed to disrupt West Nile virus (WNV) transmission and public WNV risk perception and protective behaviors throughout Northern Colorado. Colorado has some of the highest West Nile virus (WNV) case numbers in the United States each year. Current WNV control is limited to applying mosquito larvicides to water sources and insecticide spraying to control adult mosquito vectors in residential areas when the risk of WNV transmission is high, but there is limited efficacy and increasing resistance to these methods. Additionally, public health departments often provide communication about behaviors that local residents can use to protect themselves from WNV (e.g., wearing bug spray) and reduce the transmission of WNV (e.g., draining standing water), but these messages are not universally effective. Wild birds are WNV reservoirs, and the authors are developing an alternative WNV control strategy that treats birds with the drug ivermectin (IVM) to kill mosquitoes that blood feed upon them to reduce WNV transmission risk to humans. They are planning a large-scale field trial across Northern Colorado next summer to examine the efficacy of IVM-treated bird feed. To prepare for these trials, they spent this summer trapping mosquitoes in a collection of houses across Weld County to examine how people’s perceived WNV risk compared to their actual risk using vector index calculations. These preliminary data will give an insight into people’s WNV risk perceptions and protective behaviors, how these correlate with WNV risk, and how these perceptions and behaviors change in response to mosquito field work. This will help to better evaluate both potential confounders in future IVM field trials as well as consider improved WNV public health education and messaging. This study was funded by NIH/NIAID grant 5306136. All studies using human subjects or tissue samples have been either approved or deemed nonhuman subject research by the Institutional Review board of Colorado State University.

Elizabeth Walsh, Tran Zen Torres, Brian Prince, and Claudia Rückert (Dept. of Biochemistry, University of Nevada Reno) presented their research about defining the role of antiviral proteins in *Culex quinquefasciatus* mosquitoes. Culex spp. mosquitoes transmit several pathogens that threaten public health, including West Nile virus. Identifying antiviral factors in Culex spp. mosquitoes will aid virus control strategies. Mosquitoes rely on RNAi pathways to control viral replication; however, little is known about the antiviral role of RNAi proteins in *Culex quinquefasciatus* mosquitoes. Dicer-2 and Argonaute-2 are key members of the siRNA pathway and known antiviral proteins in *Aedes aegypti*. While Piwi4, a member of the piRNA pathway, is antiviral against several arboviruses in *Ae. aegypti*, this antiviral effect may be independent of the piRNA pathway. Additionally, in *Ae. aegypti*, the RNA-binding protein aBRAVO interacts with Piwi4, Dicer-2, and Argonaute-2 and is antiviral. Notably, aBRAVO is mosquito-specific, indicating a novel antiviral mechanism. This research aimed to elucidate the role of antiviral proteins in *Cx. quinquefasciatus*. The authors examined the effects of RNAi proteins, Dicer-2, Piwi4, and Argonaute-2, as well as aBRAVO, on arbovirus infection in *Cx. quinquefasciatus* cells. Silencing Dicer-2, Piwi4, and Argonatue-2 increased the virus replication of the negative-sense RNA virus La Crosse encephalitis virus (LACV). The authors found that aBRAVO increases the virus replication of LACV and also has an antiviral effect against the positive-sense RNA virus Usutu virus. Additionally, virus infection significantly increased aBRAVO expression in *Cx. quinquefasciatus* cells. These findings point to aBRAVO’s broad antiviral activity. Overall, the knowledge of antiviral proteins in *Cx. quinquefasciatus* mosquitoes was expanded. The findings underscore the need for further research to understand the complex interactions of RNAi pathways and their antiviral effects in *Culex* spp. mosquitoes. This study was funded by INBRE, National Institute of General Medical Sciences (GM103440 and GM104944), and USDA Hatch grant NEV00389. No animal or human studies were performed.

Kaitlynn A. Williams, along with Emily Gallichotte and Gregory Ebel (Department of Microbiology, Immunology, and Pathology, Colorado State University, Fort Collins, CO, USA), presented her research on the impact of temperature on the replication, dissemination, and transmission of yellow fever 17D vaccine in *Aedes aegypti*. Yellow fever virus is mainly transmitted to humans by *Aedes* species mosquitoes. Climate change projections predict dramatic range expansions for these mosquitoes and the pathogens they carry. Thus, yellow fever virus (YFV) may spread to regions with minimal vaccination rates. Over the past six years, yellow fever outbreaks in endemic regions have occurred due to a variety of factors, including poor vaccine coverage and several other factors. YFV-17D is a highly efficacious and safe live-attenuated vaccine used to prevent outbreaks in endemic areas. Previous work has shown that YFV-17D replicates in the midgut of *Aedes aegypti* mosquitoes but disseminates poorly out of the midgut compared to wild-type YFV strains. It has been shown for other arboviruses that increased temperature can increase infection, dissemination and transmission rates, and levels of virus within mosquito tissues (i.e., their competence for a given pathogen). The degree to which increased temperatures may alter the vector competence (VC) of *Aedes* mosquitoes for the attenuated YFV-17D has not been determined. The authors exposed *Ae. Aegypti* mosquitoes to YFV-17D, held them at an increased temperature (34 °C), then assessed VC and dissemination rates using qRT-PCR. Their results suggest enhanced escape from the midgut on days 7 and 14 post-infection compared to standard temperatures (29 °C). Ongoing studies will further explore the temperature dependence of VC, specifically dissemination and transmission rates for YFV-17D using qRT-PCR, plaque assays, and sequencing, to determine whether viral genetic changes are associated with midgut escape. This study was funded by National Science Foundation BII- 2,213,854, and no animal or human studies were performed.

### 2.5. Molecular Foundations of Viral Interactions

Grace Campagnola and Olve Peersen (Department of Biochemistry and Molecular Biology, Colorado State University, Fort Collins, CO, USA) presented their research on the co-folding of poliovirus P3 polyprotein precursors. Positive-strand RNA viruses use long open reading frames to express large polyproteins that are processed into individual proteins by viral proteases. Polyprotein processing is highly regulated and yields intermediate species with different functions than the fully processed proteins, increasing the biochemical diversity of the compact viral genome while also presenting challenges in that proteins must remain stably folded in multiple contexts. The authors used circular dichroism spectroscopy and single-molecule microscopy to examine the solution structure and self-association of the poliovirus P3 region protein composed of membrane binding 3A, RNA priming 3B (VPg), 3C^pro^ protease, and 3D^pol^ RNA-dependent RNA polymerase proteins. The data indicated that co-folding interactions within the 3ABC segment stabilizes the conformational state of the 3C protease region, and this stabilization requires the full-length 3A and 3B proteins. Enzymatic activity assays showed that 3ABC is also an active protease and it cleaves peptide substrates at rates comparable to 3C^pro^. The cleavage of a larger polyprotein substrate is stimulated by the addition of RNA, and 3ABC^pro^ becomes 20-fold more active than 3C^pro^ in the presence of stoichiometric amounts of viral cre RNA. The data suggest that co-folding within the 3ABC region results in a protease that can be highly activated toward certain cleavage sites by localization to specific RNA elements within the viral replication center, providing a mechanism for regulating viral polyprotein processing. Funding was provided by NIH, and no human or animal studies were performed.

Samantha J. Courtney presented her work with Rebekah J. McMinn and Sam R. Telford III of the Department of Microbiology, Immunology, and Pathology, Colorado State University, on disease phenotypes associated with the geographical structure of deer tick virus in the United States. Deer tick virus (DTV) is an emerging tick-borne flavivirus that can cause severe neurologic disease in humans, including encephalitis and meningitis. DTV is one of two genetically and ecologically distinct lineages of Powassan virus (POWV). POWV lineage I is associated with tick species that rarely bite humans; however, DTV (POWV lineage II) poses a significant threat to human health due to its association with black-legged ticks (*Ixodes scapularis*) which display aggressive human-biting behavior. A recent phylodynamic study of DTV in North America showed that there is a geographical structuring of Northeastern and Midwestern DTV clades. The extent that this phylogeographic structure is associated with phenotypes is unclear. Therefore, the authors sought to determine whether geographically and genetically defined isolates of DTV differ in mouse pathogenesis. C57BL/6 mice display human-like symptoms associated with DTV such as hemiplegia, muscle weakness, ataxia, tremors, and sometimes death. To determine any differences in morbidity and mortality, they inoculated C57BL/6 mice with 10^3^ PFU DTV isolated from *Ixodes scapularis* ticks in several locations in Wisconsin, inland New York, and coastal New York. Notably, there was a distinct difference in mortality with an inland New York DTV isolate (Saratoga Springs, NY, USA) compared to a coastal New York DTV isolate (Connetquot, NY), where the probability of survival was 30% higher for mice infected with the coastal New York isolate. They are currently repeating this study to confirm these results. Future studies will determine the role of pathogenesis for the phenotypes associated with coastal New York DTV. All animal studies were performed following guidelines and protocols approved by the Institutional Animal Care and Use Committee of Colorado State University. This work was funded by NIH grant 5RO1AI137424-04.

Emily A. Fitzmeyer, with Emily N. Gallichotte, James Weger-Lucarelli, Marylee L. Kapuscinski, Zaid Abdo, Kyra Pyron, Michael Young, and Gregory D. Ebel of the Department of Microbiology, Immunology, and Pathology at Colorado State University, presented her work on species-dependent population bottlenecks and their role in the loss of West Nile virus genetic diversity during mosquito infection. Each step during arthropod infection constitutes a physiological barrier to virus transmission. These barriers impose stochastic reductions on arbovirus populations, frequently termed bottlenecks. In vectors of West Nile virus, the main bottlenecks occur during the infection of and escape from the midgut and salivary glands. The severity of these bottlenecks varies by tissue and potentially mosquito species. Arboviruses such as WNV are maintained in nature by multiple mosquito species with varying levels of vector competence (VC, the efficiency of pathogen transmission). However, the extent to which population bottlenecks and VC are linked is poorly understood. Additionally, quantitative analyses of mosquito bottlenecks on virus population dynamics are limited. To address these knowledge gaps, the authors used molecularly barcoded WNV (bcWNV) to quantitatively measure tissue-associated population bottlenecks in *Culex tarsalis*, *Culex quinquefasciatus*, and *Aedes aegypti*—three variably competent WNV vectors. In *Aedes* mosquitoes, barcode diversity in the midgut was significantly lower compared to Culex species. Importantly, population richness and complexity did not differ significantly between salivary glands and saliva from any species. Barcode frequency in the input population was positively correlated with successful transmission in Culex; however, high frequency in the blood meal did not guarantee transmission in any species. *Cx. tarsalis* had the highest probability of transmitting rare barcodes when compared to lower-competence vectors. This work provides insight into stochastic influences on virus population dynamics during mosquito infection in vectors of varying competence and suggests that vector competence may influence the successful transmission of rare virus variants in a population. No human or animal studies were performed. The study was funded by the National Institutes of Health grant R01-AI067380, National Institutes of Health Award Number T32-AI162691, and Colorado State University’s Office of the Vice President for Research’s “Accelerating Innovations in Pandemic Disease” initiative, made possible through support from The Anschutz Foundation.

Lauren Malsick, along with Brian Geiss (Dept. of Biochemistry and Molecular Biology, Colorado State University, Fort Collins, CO, USA), presented research on the evolution of the bat RNA decay pathway during flavivirus infection. It has been observed that bats can be infected by various flaviviruses and can excrete viruses (such as Zika virus in urine), but they do not typically experience high viremia or show severe disease. The dichotomy between flavivirus infection and the lack of disease in bats has not been answered to date, and the molecular mechanisms allowing bats to avoid disease in flavivirus infection are unclear. In other mammals, the endogenous cellular RNA decay pathway has been shown to significantly influence disease pathogenesis during flavivirus infection, but how the RNA decay pathway functions in bats or how it interacts with viruses is unknown. Zika virus subgenomic flaviviral RNAs (sfRNAs) have been detected in wild Ugandan bats, indicating the natural infection of bats with Zika virus, and suggesting an interaction of Zika virus genomes with the bat XRN1 exoribonuclease enzyme (a key component of the RNA decay pathway) to generate sfRNAs. XRN1 stalls on the highly structured 3′ untranslated region (UTR) of the flavivirus genome, which results in the production of sfRNAs and influences disease in humans. The study used West Nile and Entebbe bat virus infection in a primary bat cell line, a mutation of West Nile virus sfRNA structures, and siRNA-mediated knockdowns of bat RNA decay gene homologs to investigate what role the bat RNA decay pathway plays in viral infection and determine if flavivirus sfRNAs influence replication and the cytopathic effect in bat cells. To the authors’ knowledge, this is the first investigation of the interplay between RNA viruses and the bat RNA decay pathway, which may provide valuable insights into how the RNA decay pathway (known to strongly affect viral infection in mammalian and mosquito cells) also impacts infection in bat cells. Funding was provided by an IDRRTP training grant, and no human or animal studies were performed.

Tem Morrison, along with Frances S Li, Kathryn S Carpentier, Cormac J Lucas, Bennett J Davenport, David W Hawman, Stephanie E. Ander, Heinz Feldmann, and Thomas E Morrison (^1^ Department of Immunology and Microbiology, University of Colorado School of Medicine, Aurora, CO 80045, USA; ^2^ Laboratory of Virology, Division of Intramural Research, National Institute of Allergy and Infectious Diseases, National Institutes of Health, Rocky Mountain Laboratories, Hamilton, MT 59840, USA), presented his research on the host and viral determinants of arbovirus viremia and dissemination. Arboviruses are major public health threats. The major determinants of arbovirus transmission, geographic spread, and pathogenesis are the magnitude and duration of viremia in the vertebrate host. However, the factors that dictate viremia following arbovirus infection are poorly defined. Using a mouse model, the authors found that a panel of arboviruses display distinct clearance kinetics from the circulation including swift, slow, and resistant. Moreover, for several different alphaviruses, bunyaviruses, and flaviviruses, they found that clearance requires blood-exposed phagocytes. Mechanistically, they determined that multiple arthritogenic alphaviruses, including chikungunya virus (CHIKV), are cleared efficiently from murine circulation by scavenger receptor A6 (MARCO) expressed on liver macrophages. The MARCO-dependent clearance of these viruses is contingent on the presence of specific biochemical features of the virion surface E2 and E1 glycoproteins. In contrast, while the clearance of dengue virus (DENV) and eastern equine encephalitis virus (EEEV) requires phagocytic cells, distinct pathways including mannose binding lectin (MBL) and glycosaminoglycans (GAGs), respectively, promote virion clearance. Remarkably, EEEV is resistant to clearance from the circulation in avian hosts, suggesting the existence of species specificity in virion–GAG interactions. Utilizing an in vitro cell culture system, the authors of this study uncovered that the ectopic expression of murine MARCO promoted the adsorption and internalization of CHIKV particles via the MARCO scavenger receptor cysteine-rich (SRCR) domain. Evaluation of the SRCR domain of MARCO from a panel of species revealed that the MARCO SRCR domain from vertebrates develops low to no viremia, which supports CHIKV internalization, whereas those from known amplification hosts (i.e., humans, nonhuman primates) did not, indicating that, similar to GAG-EEEV interactions, the MARCO SRCR domain interacts with CHIKV particles in a species-specific manner. In support of these observations, CHIKV particles are inefficiently cleared from the blood circulation of rhesus macaques, in striking contrast with C57BL/6 mice. Finally, following subcutaneous inoculation, the authors found that arthritogenic alphavirus particles drain via the lymph and are rapidly captured by MARCO^+^ lymphatic endothelial cells (LECs) in the draining lymph node (dLN), which limits viral spread to the bloodstream. These findings identified a previously unrecognized arbovirus-scavenging role for LECs. Collectively, this group’s studies are providing new insight into the virus–host interactions that influence arbovirus viremia, dissemination, and pathogenesis and determine whether a vertebrate serves as an amplification or dead-end host. This study was funded by R01 grant AI148144 and R01 grant AI141436. All animal studies were performed following guidelines and protocols approved by the Institutional Animal Care and Use Committee of the University of Colorado School of Medicine.

Matthew P. Taylor ^1^, along with Luke F. Domanico ^1^, Jake P. Fredrikson ^1^, Emma K. Loveday ^1^, and Connie Chang ^2^ (Montana State University, Bozeman MT ^1^, The Mayo Clinic, Rochester, MN ^2^) presented research on single-cell neuronal HSV-1 infection using drop-based microfluidics, revealing the heterogenous outcomes of viral replication. Herpes simplex virus-type 1 (HSV-1) is a prevalent human pathogen that infects neurons to mediate persistence and viral disease. HSV-1 infection of neurons can undergo either active lytic replication or can persist in a transcriptionally repressed, non-replicative state called latency. Understanding the conditions that promote the different outcomes of infection provides insight into HSV-1 disease. The authors sought to evaluate the outcomes of HSV-1 infection through the single-cell infection of neurons. To achieve this goal, they developed a drop-based microfluidic approach to the culture and infection of neurons. The single-cell growth of primary mouse Superior Cervical Ganglia (SCG) neurons was achieved on Matrigel microgels, which provide a solid scaffold for cellular adherence and the development of neurite extensions. The microgel-embedded cells were subsequently emulsified into aqueous drops with a dual fluorescent protein-expressing herpes simplex virus type 1 (HSV-1) that distinguishes the onset of viral gene expression and subsequent replication. It was observed that higher inoculating doses led to the earlier detection of gene expression onset and more extensive productive viral replication. Lower doses still resulted in infection, but only a few cells progressed to productive replication. A similar trend was observed for Vero cells, suggesting that the dose-dependent kinetics and replication outcomes are cell type-independent. Importantly, the authors were able to observe single-cell infection for longer observation periods in order to reveal a large population of “stalled” infections not readily observed in bulk infection models. These stalled infections may be related to the establishment of transcriptionally suppressed latent infections. Further studies are required to understand the stage where viral replication stalls and identify the cellular and viral factors that influence the outcomes of HSV infection. This study was funded by R21AI146952-01, and all animal studies were performed following guidelines and protocols approved by the Institutional Animal Care and Use Committee of Montana State University.

### 2.6. Understanding Viral Immunology and Vaccines

Charlotte Anderson ^1^ presented her work alongside Alaura Hoag ^1^, Amy Aspelund ^1^, Markus Wolschek ^2^, Manfred Reiter ^1,2^, and Thomas Muster ^1,2^ (^1^ Vivaldi Bioscience and ^2^ Blue Sky Immunotherapies) on the optimization and characterization of a live-attenuated SARS-CoV-2/influenza vaccine. SARS-CoV-2 and influenza are serious public health issues. If SARS-CoV-2 becomes endemic, annual co-epidemics of these pathogens could occur, requiring yearly vaccination against both diseases. The authors developed an intranasal vaccine to protect against both viruses. Their combined SARS-CoV-2/influenza vaccine is based on replication-deficient reassortant live-attenuated influenza vaccine (LAIV) strains lacking the gene for nonstructural protein 1 (NS1). They developed vaccine constructs engineered with the SARS-CoV-2 receptor binding domain and the influenza hemagglutinin (HA) transmembrane domain inserted in the influenza NS gene, resulting in the expression of membrane-anchored RBD. After constructing this combined vaccine strain, they induced previously described patented mutations to increase the viral titer while retaining its ability to co-express influenza and SARS-CoV-2 antigens. To ensure that the construct was stable with the included mutations, the strains were passaged ten times. A stability study was then performed on the strains, including growth curves and an RT-PCR study. Multiple assays were conducted to investigate the expression of SARS-CoV-2 RBD and HA in viruses with and without the high-growth mutations. Their growth curves and subsequent fluorescent focus assays demonstrated the viruses’ ability to grow to a high titer, while their RT-PCR and gel assays demonstrated that the RBD insertion is stable. Following this, they conducted dPCR experiments to determine the ratio of HA genes to RBD genes per mL of virus sample, and finally, they conducted several Western blots to investigate the general expressivity of those genes as proteins. This work was funded by NGN Capital LLC. No animal or human studies were performed.

Christian Cherry presented his research alongside Callie Lang, Ben Swartzwelter, Allison Vilander, and Gregg Dean (Dept. of Microbiology, Immunology, and Pathology, Colorado State University) on a Lactobacillus-expressing FliC driving local effector responses among resident immune cells. Developing effective vaccines against emerging pathogens is critical. The desirable features of a vaccine platform include rapid engineering, inexpensive manufacturing, simple storage and distribution, and the induction of robust, durable immune responses. Their group proposed the probiotic Lactobacillus acidophilus (LA) as an orally delivered mucosal vaccine platform. LA survives gastric acid and bile, accesses immune inductive sites, and activates critical pattern recognition receptors including TLR2, NOD2, and DC-SIGN. They engineered LA to express pathogen antigens and showed the induction of antigen-specific immune responses. While the mucosal immune system can induce local and systemic immunity, one major limitation of a mucosal vaccine is overcoming the tolerogenic state of the mucosal immune system. Therefore, to overcome this, their group engineered LA to express the TLR5 ligand Salmonella typhimurium flagellin protein FliC. Here, they showed the successful engineering of LA to express FliC via PCR screening and flow cytometry. Mice were vaccinated with wild-type LA and LA engineered to express FliC. They collected the small intestine, Peyer’s patches, and mesenteric lymph nodes. Following tissue digestion, they used flow cytometry to quantify macrophages, dendritic cells, and T cells within this mucosal immune system. In addition, they performed nuclear staining to determine CD4+ T cell differentiation into effector helper T cell subsets. Here, they showed that T cells within the intraepithelial layer display a marked sensitivity to vaccination, with LA-treated mice displaying increases in activation markers among CD8a+ T cells. Furthermore, treatment with adjuvanted Lactobacillus increases the recruitment of CD4+ T cells to the intraepithelial layer. This work was funded by NIAID R01 AI141604, the Young Investigator Award Center for Companion Animal Studies, and the T32 Dual-Degree Medical Scientist Training Program. All animal studies were performed following guidelines and protocols approved by the Institutional Animal Care and Use Committee of Colorado State University.

Janahan Loganathan ^2^ presented his work together with Ben Swartzwelter ^1^, Michelle Savran ^1^, Brian Foy ^1^, Allison Vilander ^1^, and Gregg Dean ^1^ (^1^ Department of Microbiology, Immunology, and Pathology and the ^2^ Department of Chemical and Biological Engineering, Colorado State University) on an oral vaccine delivery system that focused on coating bird seed with lyophilized recombinant lactobacillus acidophilus using sodium alginate. West Nile virus (WNV) is a mosquito-borne flavivirus that is widely distributed in Africa, Europe, the Middle East, North America, and West Asia. WNV is maintained in an enzootic cycle between bird reservoirs and *Culex* spp. mosquitoes. Spillover occurs when mosquitoes feed on the virus-amplifying avian host and proceed to transmit the virus to an aberrant host. Though many species are susceptible to infection, disease is most prevalent in humans and horses. Human infections are associated with a growing health and financial burden. Currently, there are no human antiviral treatments or vaccines available. Current control efforts target the vector by spraying vast areas; however, this presents potential negative health effects and collateral environmental impacts. Their overarching aim was to reduce instances of WNV in wild bird populations, thus limiting/preventing human cases, without affecting the natural ecosystem caused by chemical intervention. To achieve this, they developed recombinant Lactobacillus acidophilus (rLa) as an oral vaccine to immunize wild bird populations against WNV (see Michelle Savran’s abstract). They leveraged existing technology developed to deliver endectocides to wild birds by coating ivermectin on bird seed and millet. Currently, they are optimizing the lyophilization of their vaccine using sodium alginate as a protectant to ensure the maintenance of vaccine competence. It is anticipated that wild birds will be immunized against WNV through the consumption of vaccine-coated bird seed/millet. Funding was provided by NIH/NIAID R01AI148633. No animal or human studies were performed.

Michelle Savran shared research conducted alongside Ben Swartzwelter, Janahan Loganathan, Preston Schweiner, Kathryn Coffin, Brian Foy, Allison Vilander, and Gregg Dean (Dept. of Microbiology, Immunology, and Pathology, Colorado State University) on their combined efforts towards avian vaccination via recombinant Lactobacillus-bound bird seed to curb the spread of West Nile virus. West Nile virus (WNV) is the leading cause of domestically acquired mosquito-borne disease in the United States. Despite significant investment, no effective human WNV vaccines have been developed, so current mitigation efforts remain limited to environmentally toxic insecticidal sprays. While humans and other animals can develop disease, they are dead-end hosts because they do not develop high enough viremia to infect other mosquitoes. Instead, the propagation of WNV is primarily maintained between mosquitoes and birds. Together, the authors hypothesize that immunizing WNV-susceptible birds will reduce WNV transmission to mosquitoes, protecting both people and animals from infectious bites and disease. To this end, they are genetically modifying a strain of the probiotic Lactobacillus acidophilus (LA) to express WNV antigenic proteins premembrane (prM) and envelope (E). The bacteria can be administered orally and deliver intact viral protein to mucosal immune inductive sites. Immunogenicity is enhanced by the addition of a dendritic cell-targeting peptide (DCpep). Protein expression by the LA-based vaccine (rLA-WNV) will be assessed by Western blot and flow cytometry. Immunogenicity will be measured by vaccinating chickens, assessing the development of anti-WNV antibodies, and measuring viremias following WNV challenge. rLA-WNV will then be bound to bird seed for wild bird consumption. They selected this strategy because, first, it is only practical to immunize wild birds orally with food baits in WNV-endemic areas, and second, LA can be lyophilized, allowing for preservation and binding to bird seed. The strategy, if successful, will result in an innovative and cost-effective strategy for the control of vector-borne disease. Funding was provided by 5R01AI148633-04. All animal studies were performed following guidelines and protocols approved by the Institutional Animal Care and Use Committee of Colorado State University.

Tony Schountz ^1^ presented his data obtained alongside Bradly Burke ^1^, Shijun Zhan ^1^, Savannah M Rocha ^2^, Clara Reasoner ^1^, Amin Addetia ^3^, Phillida Charley ^1^, Ronald B Tjalkens ^2^, David Veesler ^3^, and Tawfik Aboellail ^1^ (^1^ Department of Microbiology, Immunology, and Pathology, Colorado State University; ^2^ Department of Environmental and Radiological Sciences, Colorado State University; and the ^3^ Dept. of Biochemistry, University of Washington) on the regulatory T cell-like response in human ACE2-transduced Jamaican fruit bats infected with SARS-CoV-2. Insectivorous Old World horseshoe bats were the likely source of the ancestral SARS-CoV-2 prior to its spillover into humans. Natural coronavirus infections of bats are principally confined to the intestines, suggesting fecal–oral transmission; however, little is known about infections of bats. Jamaican fruit bats (*Artibeus jamaicensis*) were challenged with SARS-CoV-2, and infection was confined to the intestine for only a few days and without visible signs of disease or seroconversion. Nucleocapsid antigen was detected in epithelial cells and mononuclear cells of the lamina propria and Peyer’s patches, but with no evidence of infection of other tissues. The expression of ACE2 was low in the lungs, which may account for the lack of pulmonary infection. The bats were then intranasally inoculated with a replication-defective adenovirus encoding human ACE2 and challenged with SARS-CoV-2 five days later. Viral antigen was prominent in the lungs for up to 14 days, with a loss of pulmonary cellularity during this time; however, the bats did not exhibit weight loss or visible signs of disease. From day 7, the bats had low-to-moderate IgG antibody titers to spike protein by ELISA, and one bat on day 10 produced neutralizing antibodies by a VSV pseudotype virus assay. CD4^+^ helper T cells became activated upon ex vivo recall stimulation with a nucleocapsid peptide library, and these cells expressed elevated mRNA levels of the regulatory T cell cytokines interleukin-10 and transforming growth factor-β. Collectively, these data show that Jamaican fruit bats are of low susceptibility to SARS-CoV-2 but that the expression of human ACE2 in their lungs leads to robust infection, with low-titer antibodies and a regulatory T cell-like response that may explain the lack of prominent inflammation in the lungs. This model will permit the examination of SARS-CoV-2 infection in bats and how bats’ innate and adaptive immune responses engage the virus without overt clinical disease. All animal studies were performed following guidelines and protocols approved by the Institutional Animal Care and Use Committee of Colorado State University. Funding for this study was provided through grants from the National Institute of Allergy and Infectious Diseases (NIAID) R01 AI140442 (TS, SRW); the National Science Foundation (2033260, TS; 2020297257, BB); the National Bio and Agro-Defense Facility (NBAF) Transition Fund from the State of Kansas (JAR); the MCB Core of the Center on Emerging and Zoonotic Infectious Diseases (CEZID) of the National Institutes of General Medical Sciences under award number P20GM130448 (JAR); the NIAID Centers of Excellence for Influenza Research and Surveillance under contract number HHSN 272201400006C (JAR) and the NIAID supported Center of Excellence for Influenza Research and Response (CEIRR) under contract number 75N93021C00016 (JAR), NIAID DP1AI158186, and 75N93022C00036 to DV; the National Institute of Health Cellular and Molecular Biology Training Grant T32GM007270 to AA; a Pew Biomedical Scholars Award (DV); an Investigators in the Pathogenesis of Infectious Disease award from the Burroughs Wellcome Fund (DV); and Fast Grants (DV).

Chasity E. Trammell presented their joint work with Brian J. Geiss and Gregory D. Ebel (Dept. of Microbiology, Immunology, and Pathology, Colorado State University) on the generation of broad-spectrum vaccine platforms against tick-borne flaviviruses through ancestral sequence reconstruction. Arboviruses pose a significant health threat, and there is a significant need to develop more efficient therapeutics against these pathogens. However, most developed vaccines target a specific viral species, requiring new vaccines to be developed against each pathogen. The development of vaccines that provide broad protection against a range of related viruses has the potential of reducing disease burden against known and unknown viral pathogens. In this study, they are developing novel vaccine platforms that can provide protection against a range of tick-borne flaviviruses (TBFs) that either have no or limited treatment options available. Their goal is to utilize ancestral sequence reconstruction (ASR) of viral envelope (E) and NS1 proteins of the TBF common ancestor and design vaccine candidates that express these ancestral antigens. They showed that the ASR of TBF E and NS1 has a high degree of similarity to various modern-day flaviviruses. Additionally, they also showed that they can successfully engineer constructs expressing ancestral sequences into attenuated vaccine platforms, including yellow fever virus, Sindbis virus, and deer tick virus. From these, they will generate vaccine stocks and assess replication and antigen expression. They predict that generated immune responses to ancestral antigens will provide a level of protection against multiple modern-day TBFs. The results of this study will contribute to the development of a much-needed vaccine that could be used in TBF-endemic areas. Additionally, this study will be the first to demonstrate the viability of ASR in generating broad-spectrum vaccines that could lead to the development of vaccines against other pathogens. Funding for this work was provided from the US Department of Defense (DoD) Award W81XWH-22-1-0905 and TB210011. No animal or human studies were performed.

Joseph Westrich, along with Erin McNulty, Molly Carpenter, Burton M, Audrey Sandoval, Christie Mayo, and Candace Mathiason (Department of Microbiology, Immunology, and Pathology, Colorado State University), presented research on determining longitudinal viral progression and immunological responses to Bluetongue virus in experimentally infected ruminants. Bluetongue virus (BTV) is a prevalent arthropod-borne pathogen that infects ruminant species worldwide. The severity of BTV infections ranges from asymptomatic to lethal, with the most severe cases succumbing to disease within one week. The mechanisms that determine the severity of the infection remain largely unknown. The immune response to BTV infection is thought to both contribute to the propagation of disease as well as be critical in the ultimate resolution of infection. Much of the cellular and cytokine response remains poorly understood due to limited reagent availability for the natural host species. To bridge this gap in knowledge of the role the immune response plays in BTV infection, the authors infected cohorts of sheep and muntjac with two different serotypes of BTV, BTV-10 and BTV-17. Both serotypes are endemic in the US. Interestingly, the two serotypes showed highly similar progression between the inoculated cohorts of sheep. Viremia was monitored by RT-qPCR using BTV-specific primers on intermittent blood draws. Immune cells and cytokines were evaluated by traditional flow cytometry, RNA flow cytometry, RT-qPCR, and/or fluorescent-based antibody arrays. Some potential species-specific differences, specifically in the timing of immune response and viral titers, were observed. Circulating virus was observed as early as 3 days post-inoculation (dpi) and remained detectable for the remainder of the study (24 dpi). A distinct pan-leukopenia was observed between 8 and 14 dpi that rebounded to mock-inoculated control levels at 17 dpi. The authors observed an increased expression of pro-inflammatory cytokines after 8 dpi, notably the pro-inflammatory cytokine CXCL10. Taken together, they established a model of BTV infection in two separate ruminant species and successfully monitored the longitudinal immunological response and viral progression using a combination of traditional methods and cutting-edge technology. This study was funded by the Colorado Research Council of Colorado State University’s College Veterinary Medicine and Biomedical Science and their Department of Microbiology, Immunology, and Pathology. All animal studies were performed following guidelines and protocols approved by the Institutional Animal Care and Use Committee of Colorado State University.

## Data Availability

No new data were created or analyzed in this study. Data sharing is not applicable to this article.

